# Genome guided, organ-specific transcriptome assembly of the European flounder (*P. flesus*) from the Baltic Sea

**DOI:** 10.1038/s41597-024-04004-6

**Published:** 2024-10-30

**Authors:** Konrad Pomianowski, Ewa Kulczykowska, Artur Burzyński

**Affiliations:** 1 Department of Genetics and Marine Biotechnology, Institute of Oceanology, Polish Academy of Sciences, Powstańców Warszawy 55 Str., 81-712 Sopot, Poland; 2 Present Address: Department of Genetics and Marine Biotechnology, Institute of Oceanology, Polish Academy of Sciences, Powstańców Warszawy 55 Str., 81-712 Sopot, Poland

**Keywords:** Transcriptomics, Sequence annotation

## Abstract

Although the European flounder is frequently used in research and has economic importance, there is still lack of comprehensive transcriptome data for this species. In the present research we show RNA-Seq data from ten selected organs of *P. flesus* female inhabiting brackish waters of the Gulf of Gdańsk (southern Baltic Sea). High throughput Next Generation Sequencing technology NovaSeq 6000 was used to generate 500 M sequencing reads. These were mapped against European flounder reference genome and reads extracted from the mapping were assembled producing 61k reliable contigs. Gene ontology (GO) terms were assigned to the majority of annotated contigs/unigenes based on the results of PFAM, PANTHER, UniProt and InterPro protein databases searches. BUSCOs statistics for eukaryota, metazoa, vertebrata and actinopterygii databases showed that the reported transcriptome represents a high level of completeness. The data set can be successfully used as a tool in design of experiments from various research fields including biology, aquaculture and toxicology.

## Background & summary

There are many environmental factors that interfere with physiological system controlling homeostasis in aquatic organisms, including fishes. A specific optimum condition (physical and chemical) of the internal environment is crucial for proper functioning of cells, tissues, organs and the whole organism in changing environmental conditions. The physiological system is made up of numerous control systems responsible for maintaining a state of relative equilibrium (a steady state condition) of the organism even after undergoing significant external fluctuations. Effects of external changes on organisms are usually complicated and thus require extensive studies and diverse approaches, including modern molecular research.

Flatfish species such as the European flounder (*Platichthys flesus*) are successfully used in studies of fish adaptation to different environmental conditions such as changed water salinity and temperature^1,2^ as well as environmental pollution^3^. There are many examples of research with the European flounder as a model fish species, nevertheless, available RNA-seq data deposited among NCBI bioprojects (PRJEB53201, PRJEB59805, PRJNA276828, PRJNA799325)^4–7^ are the results of sequencing of a single (liver) or mixed tissue samples, in the most cases without assembly and annotations. The reference genome of *P. flessus* is available^8^, but the lack of organ specific transcriptomic data based on this reference genome hampers comparative molecular approaches to research questions. Moreover, there are several other reasons why the assembled transcriptome sequence is important from the researcher’s point of view. Firstly, the European flounder is commercially one of the most important flatfishes in the Baltic Sea^9,10^, secondly, this marine teleost lives, breeds and prospers in water of different salinities, fresh, brackish (Baltic Sea) and ocean^11^. This exceptional adaptability makes the European flounder an excellent model to study osmoregulation processes, but not only. *P. flesus* is widely used in toxicological studies and as an environmental bio-indicator. Therefore, the influence of changing environmental conditions on fish physiology are in the spotlight of marine and freshwater biologists and ecologists, aquaculturiests, toxicologists and climatologists^2,3,12–17^. Moreover, the flounder can be easily kept in the laboratory so that many research groups are carrying out experiments on this species. Consequently, there are many studies concerning mechanisms of adaptation to different salinities, reproductive success about salinity, metamorphosis, response to environmental pollution and gene flow between populations that are done just on flounder^1,18–21^.

On the other hand, with the increasing number of an annotated reference genome assemblies, transcriptome assemblies are becoming less interest nowadays. However, high quality transcriptome data of the European flounder can still provide tools and a starting point for tracking of the molecular mechanisms underlying the disruption of homeostasis and reproductive success of fishes and therefore are important not only from the basic science point of view but also have implications for fisheries and resource management.

In this study, we report transcriptomic data obtained from ten organs of the European flounder female from the Gulf of Gdańsk (southern Baltic Sea). We used reference genomic data of *P. flesus*^8^ to guide the assembly. BUSCOs statistics for eukaryote, metazoa and actinopterygii databases suggest that the reported transcriptome represents a high level of completeness and the assembly extends the NCBI annotations, validates genes which did not have previous biological support (supplementary Table S1). The quality and usability of this data set has already been confirmed^22^. Data set that is presented here was also used to design of experiments that require using molecular biology techniques (e.g. RT-qPCR, microarray) in tracking gene expression changes^23^.

## Methods

One European flounder (*Platichthys flesus*) female was collected in the Gulf of Gdańsk (Poland) in December 2018, transported to the Institute of Oceanology PAS and kept in 200-L aerated aquarium (in salinity 7 ppt, 8 ± 0.2 °C water temperature and 8 L:16D natural photoperiod) two weeks before sampling. Whole organs: eyeball, brain, and approximately 5 × 5-mm samples of intestines, spleen, heart, liver, head kidney, gonads as well as skin from upper and bottom part of the fish were dissected starting from 10 p.m. During sampling all tissues were immediately transferred to the cooling bath composed of dry ice and 95% EtOH mixture and stored at −70 °C until RNA extraction.

### RNA extraction and sequencing

Total RNA was purified with GenElute™ Mammalian Total RNA Miniprep Kit (RTN70, Sigma-Aldrich, St. Louis, MO, USA) with minor modifications. Samples were homogenized immediately after taking from −70 °C on ice in 500 µL of Lysis Solution supplemented with proteinase K (0.6 mg/mL, E4350, EURx, Poland) and 2-mercaptoethanol (0.6% v/v, M3148, Sigma-Aldrich, St. Louis, MO, USA) and incubated for 30 min at 55 °C. RNA integrity was determined before sequencing (Macrogen Inc. Korea). All samples passed the quality criteria (see quality check report available as supplementary material). Equal amounts of RNA isolates were sequenced on an Illumina NovaSeq 6000 platform (TruSeq NGS library) with 150 bp paired-end run mode and 40 M reads per sample throughput. Initially, a total of more than 5.1 × 10^8^ raw PE reads were obtained from all libraries (Table S2, supplementary file contains raw sequencing report). Then, after filtering by removal of adaptor sequences, contaminated and poor-quality reads we obtained approximately 75Gbp of clean data (Q20 bases > 99%).

### Genome guided assembly of fish transcriptome

Trinity version 2.15.1^24^ assembler with default parameters was used to obtain genome guided assembly of the combined reads from all samples. First, the clean reads were mapped against the genome using STAR (version 2.7.9)^25^, according to the Trinity manual, then the reads were extracted from the mapping and assembled in Trinity. The read mapping rate per sample is available as supplementary Table S3. There were 351599 contigs in this initial, highly redundant assembly. The redundancies were reduced by applying CD-HIT-EST program^26^, with parameter -c 0.98 and by filtering off very poorly expressed transcripts (TPM < 1.0), as recommended by the Trinity manual. Contigs matching likely contaminants (similarity > 85% to inconsistent taxa) were removed. The final filtered assembly consisted of 61183 contigs (or Trinity isoforms) in 30860 unigenes (defined as Trinity “groups”). The average GC contents of assembled transcripts was 47.7% and N50 length was 2728 bp.

We applied version 5.7.0 of the BUSCO pipeline^27^, with odb10 database, to assess the completeness of the assembly and consistently found more than 70–80% of the representative BUSCOs in the reported transcriptome (Table 1). For the metazoa and actinopterygii BUSCOs the statistics were also very good, suggesting that the transcriptome represents a rather high level of completeness. The relatively large fraction of duplicated BUSCO for all databases suggests, that the number of alternatively assembled isoforms is still high in the final assembly.

### Functional annotation of transcriptome

To annotate the assembled unigenes, we searched for the homologous sequences of all isoforms in three protein databases: UniRef90 (2024/04 release)^28^, PFAM (release 37)^29^ and PANTHER (release 19.0)^30^. All databases were searched on a local high performance computer cluster. The two databases containing protein profiles (PANTHER and PFAM) were searched with hmmer (hmmer.org, version 3.4), UniRef90 was searched with Mmseqs2^31^ and the results were integrated into congruent annotation using tritoconstrictor available on GitHub^1^. This tool uses the following heuristics. Database hits with bitscore higher than 20 were subject to hierarchical clustering and only the top hits were used in the final annotation. Gene ontology (GO) terms were assigned to those annotated unigenes based on the current official release^32^ using mapping files provided by UniRef and PANTHER. Additionally, based on PFAM and PANTHER signatures, some unigenes were classified according to InterPro system^33^, and GO terms for these unigenes were also integrated. Majority of unigenes from the reference assembly were assigned some GO terms (Table 2).

**Table 1:** BUSCO scores.

Database (number of BUSCOS)	Complete	Single copy	Duplicate	Fragmented
Eukaryota (255)	209	94	115	12
Metazoa (954)	774	364	410	51
Vertebrata (3354)	2505	1297	1208	201
Actinopterygii (3640)	2650	1425	1225	127

Number of BUSCOS in each database, and the number of compele (single copy plus duplicated), as well as fragmented BUSCOs found in the reference transcriptome.

**Table 2:** List of raw reads and per sample assembly statistics.

BioSample	Data	ID	Organ	Number of raw reads	Effective library size	Normalized expression	Gene Ontology term assignment				
							Transcript number	Unigene number	Normalized expression		Representative terms (average unigene expression)
SAMN15147518	SRR11936462	G	gonad	51,105,632	1,908,614	523,444	25980	16202	433873	82%	biological_process/reproductive process (88)
SAMN15147519	SRR11936461	jel	intestine	50,670,370	1,070,765	933,160	31940	17336	675065	72%	molecular_function/ferric iron binding (870)
SAMN15147520	SRR11936460	Mozg	brain	56,467,492	1,309,984	762,752	32923	18337	548605	72%	molecular_function/structural constituent of myelin sheath (601)
SAMN15147521	SRR11936459	Ngl	head kidney	40,697,110	1,327,568	752,763	31580	17463	478840	63%	biological_process/antigen processing and presentation (511)
SAMN15147522	SRR11936458	oko	eye	49,715,066	1,060,061	942,593	32486	18237	688716	73%	cellular_component/photoreceptor outer segment (2027)
SAMN15147523	SRR11936457	se	heart	65,349,164	797,850	1,252,196	31189	16992	1040640	78%	biological_process/actin filament-based movement (2747)
SAMN15147524	SRR11936456	skd	skin, lower body part	46,383,752	523,601	1,907,930	27491	16860	1410357	83%	cellular_component/myosin complex (2536)
SAMN15147525	SRR11936455	skg	skin, upper body part	51,767,234	1,161,900	859,983	32411	17733	585514	68%	cellular_component/protein-lipid complex (413)
SAMN15147526	SRR11936454	sled	spleen	53,686,144	1,298,240	769,680	30738	17080	534613	69%	molecular_function/oxygen carrier activity (5066)
SAMN15147527	SRR11936453	W	liver	44,331,924	417,704	2,392,067	25433	15414	2117410	89%	molecular_function/lipid transporter activity (5708)

## Data Records

The sequencing and assembly data of transcriptome for all samples were deposited into public repositories: The transcriptome sequencing data generated in this work were deposited as SRR11936453-SRR11936462 in NCBI Sequence Read Archive^34^. The assembly was deposited in Transcriptome Shotgun Assembly and is linked to BioProject accession number PRJNA637628 in the NCBI BioProject database^35^. This Transcriptome Shotgun Assembly project has been deposited at DDBJ/ENA/GenBank under the accession GKXD00000000. The version described in this paper is the first version, GKXD01000000^36^. All the TSA records are fully annotated. Additional data, including expression profiling across samples, are available as supplementary material (supplementary Table S4.xlsx).

## Technical Validation

### RNA integrity

The transcriptome for ten  organs from one fish individual were sequenced. Before constructing RNA-Seq libraries, the concentration was measured on Epoch™ Microplate Spectrophotometer (BioTek, Winooski, USA) and the quality of total RNA was evaluated using Agilent BioAnalyzer 2010. The total amount of RNA, RNA integrity number (RIN) and rRNA ratio were used to estimate the quality and concentration of RNA. Samples with a total RNA amount ranging from 1.85 to 6.36 μg, RIN 6.5 to 8.6 and rRNA ratio 1.0 to 1.5 were used to construct sequencing libraries.

### Quality filtering of Illumina sequencing raw reads

The raw sequence reads obtained from Illumina platform (Macrogen Inc. Korea) were rigorously cleaned by the following procedure. Trimmomatic (version 0.39) was used to filter out contaminating adaptor sequences and poor quality reads with the following command line options: “ILLUMINACLIP:adapters.fa:2:30:10 LEADING:10 TRAILING:10 SLIDINGWINDOW:6:20 MINLEN:55”. If any member of the pair was discarded, both pairs were discarded. The initially generated raw sequencing reads were also evaluated regarding quality distribution, GC content distribution, base composition, average quality score at each position and other metrics and showed no anomalies (the QC report from sequencing company is included as supplementary data). Trimming resulted in only marginal improvement of the already very good quality of the raw reads (Table 3).

**Table 3:** Read quality before and after filtering.

Accession in SRA		forward	Q20%	reverse	Q20%
SRR11936453	untrimmed	3296731323	98.4963	3282123912	98.0599
	trimmed	3217280035	98.6548	3207149312	98.3428
SRR11936454	untrimmed	3984625993	98.3056	3970943068	97.9681
	trimmed	3875568707	98.5117	3867811085	98.3107
SRR11936455	untrimmed	3812910303	97.5562	3788907524	96.9420
	trimmed	3724766914	97.8166	3707014595	97.3384
SRR11936456	untrimmed	3418215395	97.6083	3409695433	97.3650
	trimmed	3337852201	97.8469	3334445058	97.7298
SRR11936457	untrimmed	4856220197	98.4264	4854587498	98.3933
	trimmed	4700887233	98.6035	4704579227	98.6751
SRR11936458	untrimmed	3690272782	98.3158	3681594836	98.0846
	trimmed	3583753388	98.5352	3580951595	98.4429
SRR11936459	untrimmed	2997336511	97.5495	2982971849	97.0820
	trimmed	2925532984	97.8169	2916110861	97.4871
SRR11936460	untrimmed	4189116877	98.2601	4178717535	98.0161
	trimmed	4077458882	98.5000	4073333284	98.3820
SRR11936461	untrimmed	3765681953	98.4334	3741264360	97.7952
	trimmed	3660058384	98.6349	3641796957	98.1516
SRR11936462	untrimmed	3788896484	98.1967	3774397862	97.8210
	trimmed	3680270617	98.4697	3671972830	98.2382

Number of base pairs with Q20 in untrimmed and trimmed read sets.

**Fig. 1:**
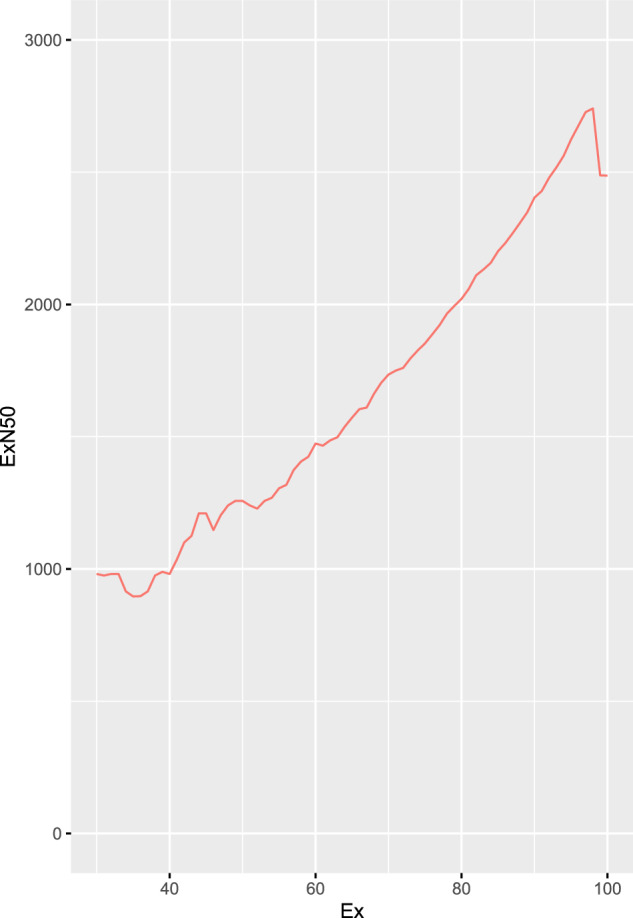
The relationship between unigene N50 (ExN50) and the percentage of scored unigenes (Ex). This figure was prepared using perl script distributed with Trinity (contig_ExN50_statistic.pl) and indicates that, taking into account the observed expression profile, the sequencing coverage was satisfactory (compare with https://raw.githubusercontent.com/wiki/trinityrnaseq/trinityrnaseq/images/Ex_vs_N50.png).

To verify if the sequencing effort was adequate, the ExN50 plot was used, as suggested by the Trinity manual (Fig. 1). In this plot, as the read depth is increased, the peak shifts towards ~90%. Clearly, adequate sequencing effort was undertaken to achieve saturation in the presented data set.

## Supplementary information

Table S1

Table S2

Table S3

Table S4

## Data Availability

^1^The code used to generate the data in the presented manuscript is fully available on GitHub: https://github.com/aburzynski/tritoconstrictor.
