# Supplementary material for: Genome guided, organ-specific transcriptome assembly of the European flounder (P. flesus) from the Baltic Sea
Source: Sci Data. 2024 Oct 30;11:1184. doi: 10.1038/s41597-024-04004-6 (PMC11525550; doi:10.1038/s41597-024-04004-6)
Supplement: Supplementary file 1 — Table S1 [file 41597_2024_4004_MOESM1_ESM.docx]

| **Gene ID** | **Gene product** | **Reference** | **Range** | **Expression profile** |
| --- | --- | --- | --- | --- |
| TRINITY_GG_631_c70_g1 | Interleukin 7 | NC_084965 | 9311990..9315471 | mostly liver |
| TRINITY_GG_145_c127_g1 | Interleukin of 15/21 family | NC_084949 | 20481361..20486077 | spleen, skin, head kidney |
| TRINITY_GG_559_c8_g1 | Hepcidin | NC_084955 | 4204446..4205172 | only liver |

Table S1. Examples of the predicted genes, corresponding to the transcripts from genome guided transcriptome assembly, but not matching any annotated genes in the reference genome (version GCF_949316205.1_fPlaFle2.1, relevant accession numbers of the chromosomes are shown in the table). The mapping of these new transcripts to the reference genome is unambiguous, at the indicated location.
