# Supplementary material for: Genome guided, organ-specific transcriptome assembly of the European flounder (P. flesus) from the Baltic Sea
Source: Sci Data. 2024 Oct 30;11:1184. doi: 10.1038/s41597-024-04004-6 (PMC11525550; doi:10.1038/s41597-024-04004-6)
Supplement: Supplementary file 2 — Table S2 [file 41597_2024_4004_MOESM2_ESM.pdf]

Research use only

Raw Data

Report

February 2019

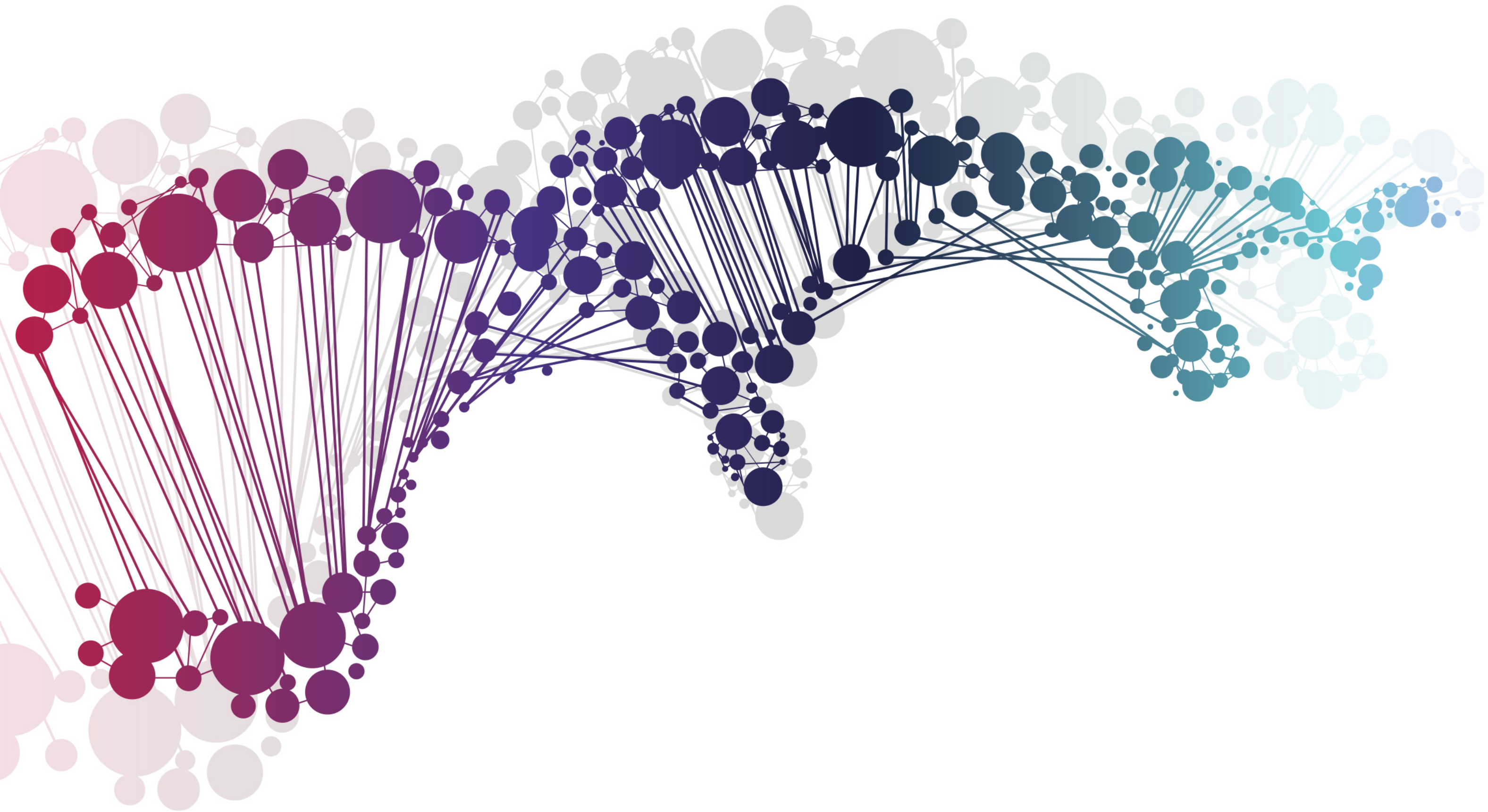

Humanizing Genomics
macrogen

COPYRIGHT © MACROGEN INC.

Project Information

Client Name	Artur Burzynski
Company / Institution	Polska Akademia Nauk
Order Number	HN00101497
Type of Read	Paired-end
Read Length	151
Number of Samples	10
Library Kit	TruSeq RNA Sample Prep Kit v2
Library Protocol	TruSeq RNA Sample Preparation v2 Guide, Part # 15026495 Rev. F
Type of Sequencer	Illumina platform

Table of Contents

Project Information 2

1. Data Download Information 4
  1. 1. Raw Data and Analysis Results 4

2. Experimental Methods and Workflow 5
  2. 1. Experiment Overview 5
  2. 2. Generation of Raw Data 6

3. Summary of Produced Data 7
  3. 1. Raw Data Statistics 7
  3. 2. Total Read Bases 8
  3. 3. Total Reads 9
  3. 4. GC/AT Content 10
  3. 5. Q20/Q30 (%) 11

4. Appendix 12
  4. 1. FAQ 12
  4. 2. FASTQ File 12
  4. 3. Phred Quality Score Chart 12

1. Data Download Information

1. 1. Raw Data and Analysis Results

Download link	File size	md5sum
Stornia_1_skd_1.fastq.gz	1.7G	d2e365221eea7c1bfa508b3006a4c2c0
Stornia_1_skd_2.fastq.gz	1.7G	a8dd767e9e21b20e1f0383d5902b879e
Stornia_1_jel_1.fastq.gz	1.7G	5db0a3c0150383edc6b9d69739e601ea
Stornia_1_jel_2.fastq.gz	1.8G	0725328399c48933266df363deb03720
Stornia_1_sled_1.fastq.gz	1.9G	30d10778273f6e4e1200cffc8a9de568
Stornia_1_sled_2.fastq.gz	1.9G	25fecd100dc841ee9d49bcab4250e84f
Stornia_1_Ngl_1.fastq.gz	1.5G	0de6d72f92608356b12ea262271a0701
Stornia_1_Ngl_2.fastq.gz	1.5G	a24fa8ffbcf0b6b3881f79ed18f9dcb9
Stornia_1_Mozg_1.fastq.gz	2.0G	3e19e22a1a144edb6d657b88b7260201
Stornia_1_Mozg_2.fastq.gz	2.0G	056d7c14c767c2bb8361bed73b0a8843
Stornia_1_skg_1.fastq.gz	1.9G	69c04b085691fc59a713f3ac34453ed9
Stornia_1_skg_2.fastq.gz	1.9G	325d822bf0000d438e1ba326dca6d3f7
Stornia_1_G_1.fastq.gz	1.8G	11ed90e6674bba061f115b1a0d1c0fb2
Stornia_1_G_2.fastq.gz	1.8G	f8c2f2231800ec0f51bf514035afd370
Stornia_1_se_1.fastq.gz	2.2G	69f94c8b170e09c3f844647bbcfc2e7e
Stornia_1_se_2.fastq.gz	2.3G	e3259e553e209bddbabca6ab0d32e87f
Stornia_1_oko_1.fastq.gz	1.7G	0993b79dd07a161efa4085446cf284a0
Stornia_1_oko_2.fastq.gz	1.8G	02f04725fb2c1343f7886871d94bd0b6
Stornia_1_W_1.fastq.gz	1.5G	9900fe3140535ceadb64aa558d575289
Stornia_1_W_2.fastq.gz	1.6G	e2dfba67778849eac49778b38ef8caf6

- fastq.gz : This is a zip file of raw data used in analysis.
- md5sum : In order to verify the integrity of files, md5sum is used. If the values of md5sum are the same, there is no forgery, modification or omission.

Your data will be retained in our server for 3 months. Should you wish to extend the retention period, please email (ngs@macrogen.com) or contact our sales team.

2. Experimental Methods and Workflow

2. 1. Experiment Overview

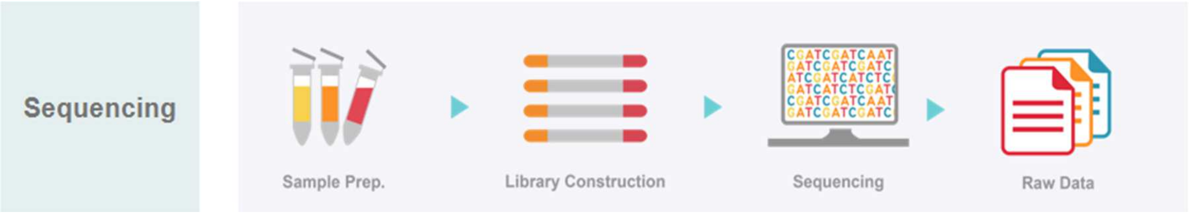

Fig1. Experiment overview

The Illumina NGS workflow includes 4 basic steps :

1) Sample Preparation

For library construction, DNA/RNA is extracted from a sample. After performing quality control (QC), qualified samples proceed to library construction.

2) Library Construction

The sequencing library is prepared by random fragmentation of the DNA or cDNA sample, followed by 5' and 3' adapter ligation. Alternatively, "tagmentation" combines the fragmentation and ligation reactions into a single step that greatly increases the efficiency of the library preparation process. Adapter-ligated fragments are then PCR amplified and gel purified.

3) Sequencing

For cluster generation, the library is loaded into a flow cell where fragments are captured on a lawn of surface-bound oligos complementary to the library adapters. Each fragment is then amplified into distinct, clonal clusters through bridge amplification. When cluster generation is complete, the templates are ready for sequencing.

Illumina SBS technology utilizes a proprietary reversible terminator-based method that detects single bases as they are incorporated into DNA template strands. As all 4 reversible, terminator-bound dNTPs are persent during each sequencing cycle, natural competition minimizes incorporation bias and greatly reduces raw error rates compared to other technologies. The result is highly accurate base-by-base sequencing that virtually eliminates sequence-context-specific errors, even within repetitive sequence regions and homopolymers.

4) Raw data

Sequencing data is converted into raw data for the analysis.

2. 2. Generation of Raw Data

The Illumina sequencer generates raw images utilizing sequencing control software for system control and base calling through an integrated primary analysis software called RTA (Real Time Analysis). The BCL (base calls) binary is converted into FASTQ utilizing illumina package bcl2fastq. Adapters are not trimmed away from the reads.

3. Summary of Produced Data

3. 1. Raw Data Statistics

The total number of bases, reads, GC (%), Q20 (%), and Q30 (%) are calculated for the 10 samples. For example, in Stornia_1_skd, 46,383,752 reads are produced, and total read bases are 7.0G bp. The GC content (%) is 51.373% and Q30 is 93.321%.

Table 1. Raw data Stats (maximum 20 samples)

Sample ID	Total read bases (bp)	Total reads	GC(%)	AT(%)	Q20(%)	Q30(%)
Stornia_1_skd	7,003,946,552	46,383,752	51.373	48.63	97.487	93.321
Stornia_1_jel	7,651,225,870	50,670,370	49.174	50.83	98.114	94.613
Stornia_1_sled	8,106,607,744	53,686,144	50.824	49.18	98.137	94.749
Stornia_1_Ngl	6,145,263,610	40,697,110	46.566	53.43	97.316	92.905
Stornia_1_Mozg	8,526,591,292	56,467,492	48.309	51.69	98.138	94.798
Stornia_1_skg	7,816,852,334	51,767,234	49.127	50.87	97.249	92.788
Stornia_1_G	7,716,950,432	51,105,632	51.314	48.69	98.009	94.569
Stornia_1_se	9,867,723,764	65,349,164	51.960	48.04	98.410	95.385
Stornia_1_oko	7,506,974,966	49,715,066	48.799	51.2	98.200	94.895
Stornia_1_W	6,694,120,524	44,331,924	51.482	48.52	98.278	94.995

- Sample ID : Sample name.
- Total read bases : Total number of bases sequenced.
- Total reads : Total number of reads. For Illumina paired-end sequencing, this value refers to the sum of read 1 and read 2.
- GC(%) : GC content.
- AT(%) : AT content.
- Q20(%) : Ratio of bases that have phred quality score of over 20.
- Q30(%) : Ratio of bases that have phred quality score of over 30.

3. 2. Total Read Bases

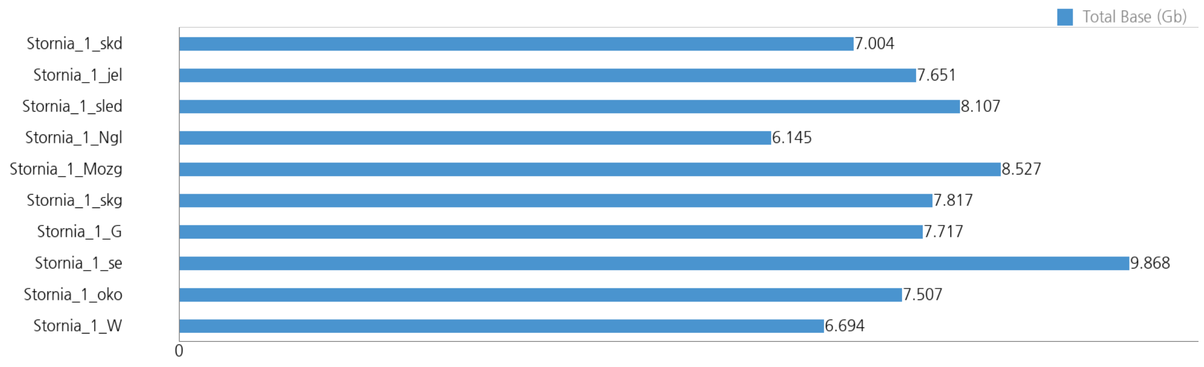

Figure 2.Throughput of Raw data

3. 3. Total Reads

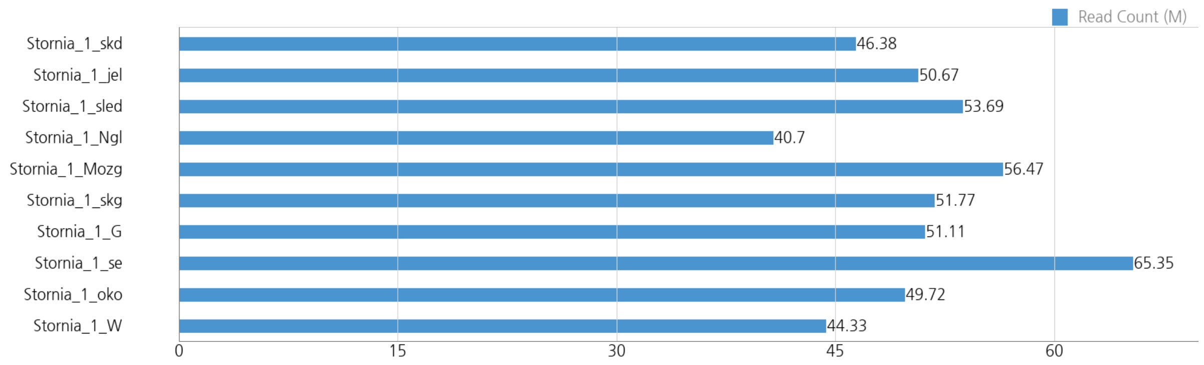

Figure 3. Total read count of Raw data

3. 4. GC/AT Content

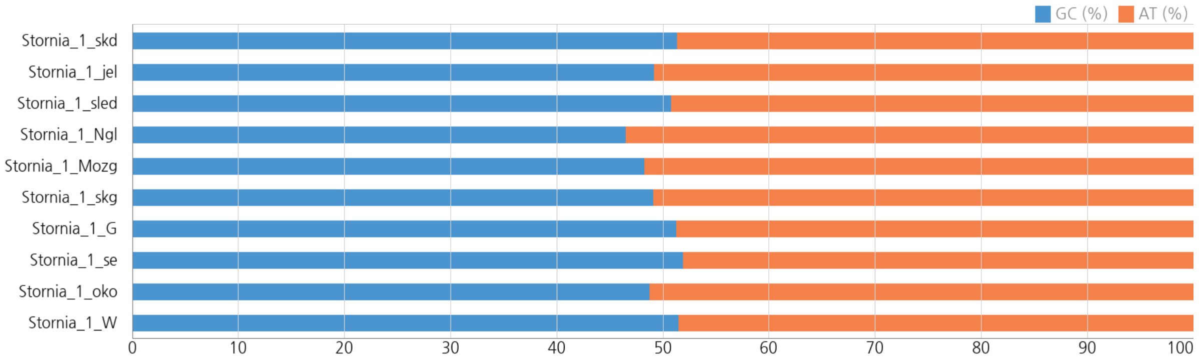

Figure 4. GC/AT Content of Raw data

3. 5. Q20/Q30 (%)

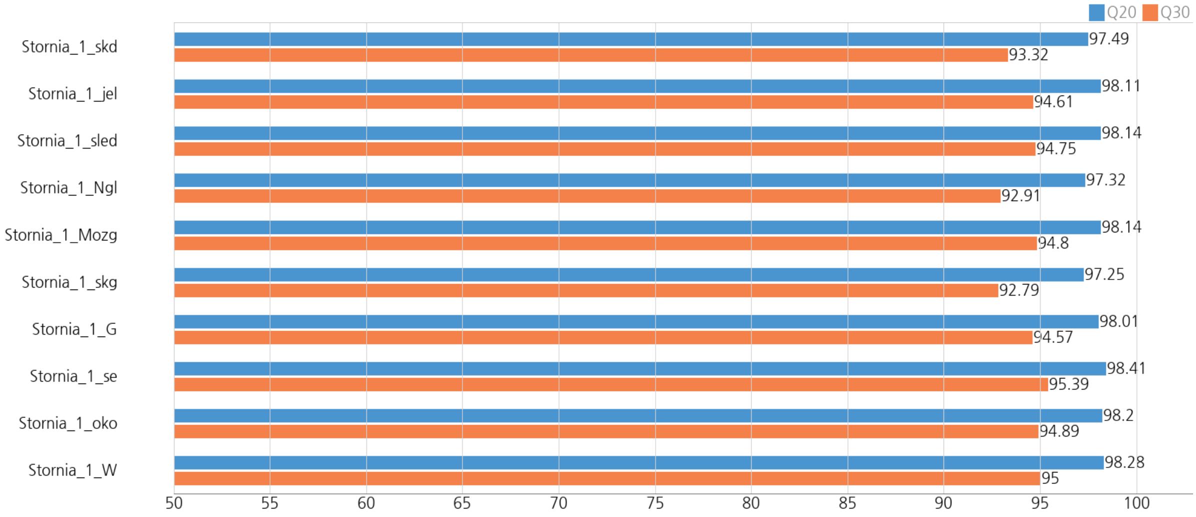

Figure 5. Q20/Q30 scores of Raw data

4. Appendix

4. 1. FAQ

Q: I want to see the produced data. How can I open the files?

A: As the large size zip files provided by our company are hard to process in the Windows environment, we highly recommend using Linux environment for a smoother operation.

4. 2. FASTQ File

Example of FASTQ

```
@HISEQ-MFG:501:HB0TFADXX:1:1101:1247:2183 1:N:0:
CTCAGCTAAATACTTTGACACCNGTANNANNNNNNNNNTNNNNNNNNNNNNN
+
@@@BDDDDHHHHFHIIIIIIII#3AC##########################
```

FASTQ file is composed of four lines.
Line 1 : ID line includes information such as flow cell lane information.
Line 2 : Sequences line.
Line 3 : Separator line (+ mark).
Line 4 : Quality values line about sequences.

4. 3. Phred Quality Score Chart

Phred quality score numerically expresses the accuracy of each nucleotide. Higher Q number signifies higher accuracy. For example, if Phred assigns a quality score of 30 to a base, the chances of having base call error are 1 in 1000.

Phred Quality Score Q is calculated with $-10\log_{10}P$, where P is probability of erroneous base call.

Quality of phred score	Probability of incorrect base call	Base call accuracy	Characters
10	1 in 10	90%	!"#$%&'()*+
20	1 in 100	99%	,-./012345
30	1 in 1000	99.9%	6789:;h=i?
40	1 in 10000	99.99%	@ABCDEFGHIJ

- Encoding : Sanger Quality (ASCII Character Code=Phred Quality Value + 33)

Humanizing Genomics
macrogen

Macrogen Inc. | 08511 10F, 254 Beotkkot-ro, Geumcheon-gu, Seoul, Rep of Korea

Contact us
Tel:+82-2-2180-7016
Site: www.macrogen.com | http://dna.macrogen.com

macrogen

Original Sample QC

General Information

Order Number	HN00101497	Name of Customer	Artur Burzynski	Date of Order	2019-01-18

Final QC Result of DNA sample(s)					
Arrival Date	Experiment Date	Sample count	Pass	Fail	Hold
N/A	N/A	N/A	N/A	N/A	N/A

Final QC Result of RNA sample(s)					
Arrival Date	Experiment Date	Sample count	Pass	Fail	Hold
2019-01-18	2019-01-21	10	7	0	3

The QC criteria refer to the specification requirements of a single run. In any cases, we may encounter the shortage of sample volume or amount due to various reasons such as a library construction failure. In these cases a request of an additional sample will be inevitable.

Therefore, we recommend double the amount to be supplied at first place to minimize any delay of the whole procedure.

*** Pass : Proceed with the library construction.**

*** Fail : Further processes are on hold until the replacement samples received.**

We do not recommend in proceeding further steps until a specific instruction was given from the client.

*** Hold : A specific instruction should be given by the client for further processing as the QC pattern may be triggered by the sample nature.**

Macrogen does not proceed the next step until we have received your permission.

As 3 ul was taken from the sample for sample (library) QC purposes, the indicated volume represents 3ul less than the total volume received.

QC Result of RNA

Arrival Date	2019-01-18	Experiment Date	2019-01-21	Tested by	CNY
Comment					

#	Sample Name	Conc. (ng/ul)	Final Volume (ul)	Total Amount (ug)	RIN	rRNA Ratio	Result*	
1	Stornia_1_oko	70.818	32	2.266	7.2	1	Pass	
2	Stornia_1_Mozg	69.945	28	1.958	8.6	1.4	Pass	
3	Stornia_1_skg	59.818	31	1.854	6.5	1	Hold	RIN
4	Stornia_1_skd	61.49	32	1.968	6.7	1	Hold	RIN
5	Stornia_1_jel	165.545	30	4.966	7.1	1.5	Pass	
6	Stornia_1_sled	75.009	28	2.1	7	1.3	Pass	
7	Stornia_1_se	77.533	32	2.481	7.6	1.3	Pass	
8	Stornia_1_W	235.823	27	6.367	7	1.6	Pass	
9	Stornia_1_Ngl	92.641	28	2.594	6.7	1.2	Hold	RIN

#	Sample Name	Conc. (ng/ul)	Final Volume (ul)	Total Amount (ug)	RIN	rRNA Ratio	Result*	
10	Stornia_1_G	167.223	27	4.515	7.7	1.4	Pass	

Experiment Condition	TapeStation RNA Screen Tape

Click to Enlarge =>1:Stornia_1_oko

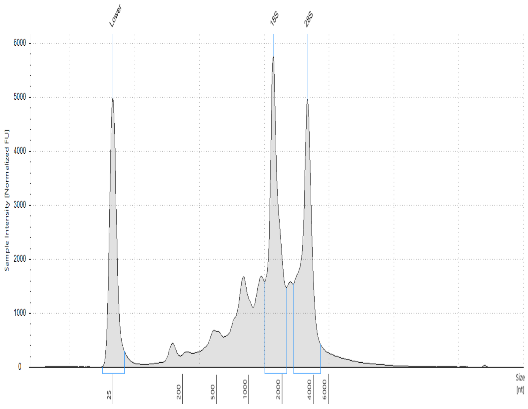

Click to Enlarge =>2:Stornia_1_Mozg

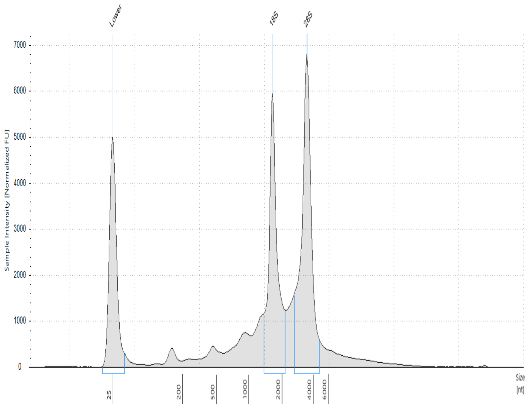

Click to Enlarge =>3:Stornia_1_skg

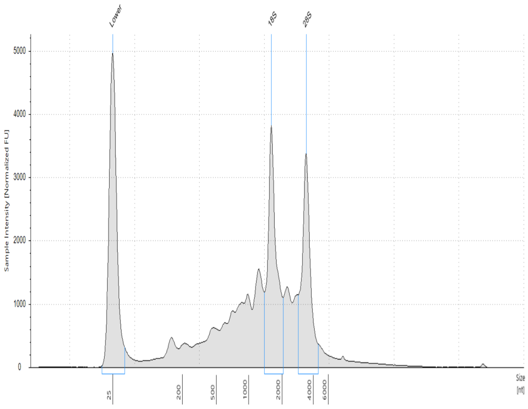

Click to Enlarge =>4:Stornia_1_skd

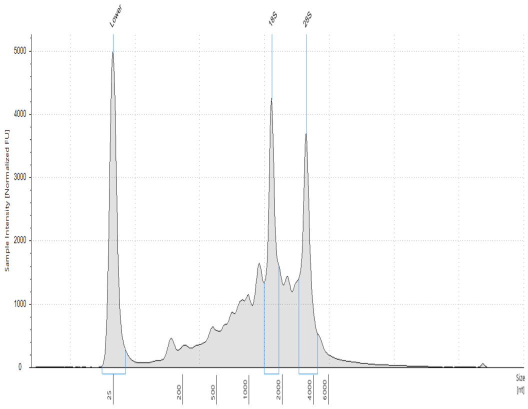

Click to Enlarge =>5:Stornia_1_jel

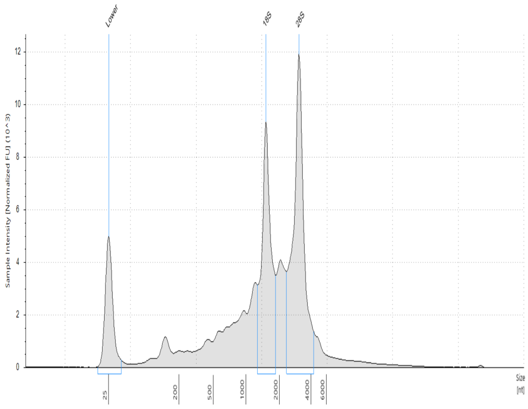

Click to Enlarge =>6:Stornia_1_sled

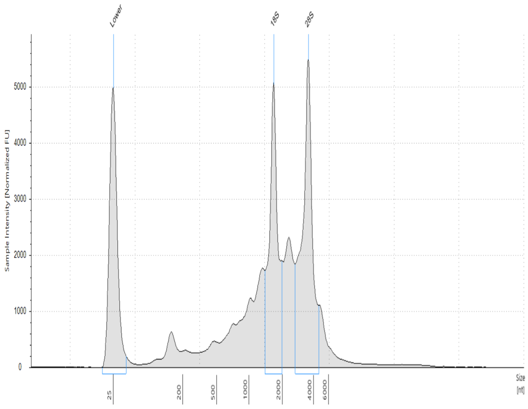

Click to Enlarge =>7:Stornia_1_se

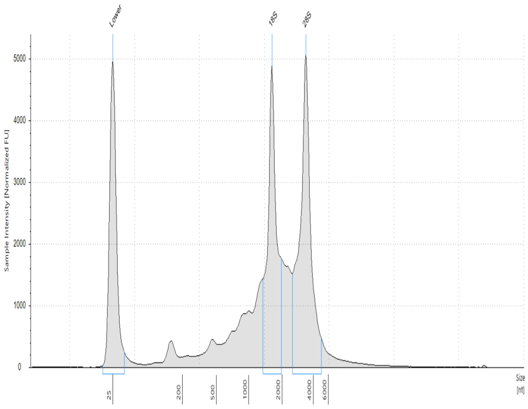

Click to Enlarge =>8:Stornia_1_W

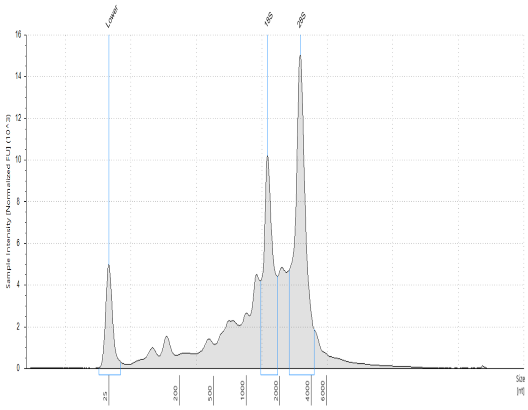

Experiment Condition	TapeStation RNA Screen Tape

Click to Enlarge =>9:Stornia_1_Ngl

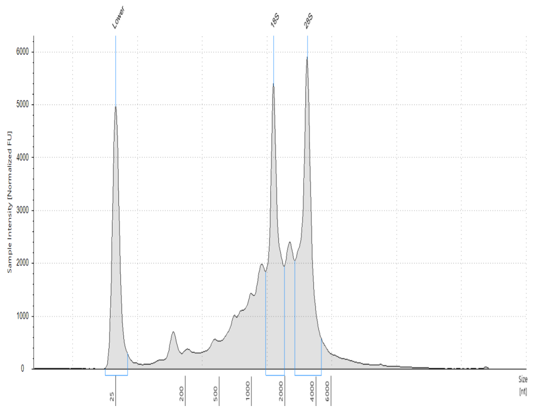

Click to Enlarge =>10:Stornia_1_G

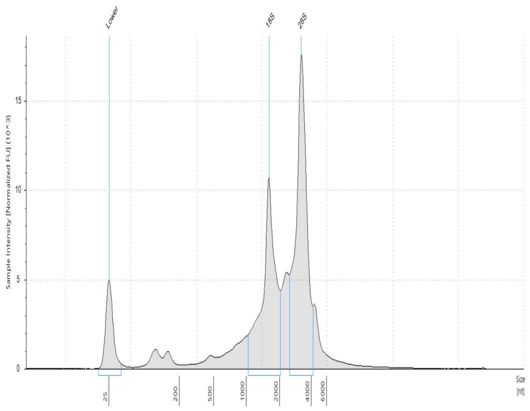

DNA QC Method

1. Quantity of DNA : Done by picogreen* method using Victor 3 fluorometry.

Macrogen quantifies the starting genomic material by a fluorescence-based quantification, rather than a UV-spectrometer-based method.
This is because fluorescence-based methods, which employ a double-stranded DNA specific dye, will specifically and accurately quantitate dsDNA even in the presence of many common contaminants. UV spectrometer methods based on 260 OD readings are prone to overestimating the DNA concentration due to the presence of RNA and other contaminants commonly found in gDNA preparations.
* Picogreen (Invitrogen, cat.#P7589)

2. Assesing the condition of the DNA : Done by gel electrophoresis method.

Gel electrophoresis is a powerful means for revealing the condition (including the presence or absence) of DNA in a sample.
Impurities, such as detergents or proteins, can be revealed by smearing of DNA bands. RNA, which interferes with 260 nm readings, is often visible at the bottom of a gel.
A ladder or smear below a band of interest may indicate nicking or other damage to DNA.

3. Size Check of DNA [Optional , Upon request, Charged]

1) DNA fragments <1kb : 2100 Bioanalyzer* is used for checking the size.
*Macrogen use DNA 1000 chip and DNA 7500chip for normal PCR product, highsensitivity chip for very small amount of DNA fragment such as ChIPed DNA.
*http://www.genomics.agilent.com/CollectionSubpage.aspx?PageType=Product&SubPageType=ProductData&PageID=1636
2) DNA fragments < 150kb : PFGE method is used for large size of DNA fragment. Please keep in mind that we condut this upon request and will be charged.

RNA QC Method

1. Quality & Quantity Check of RNA : 2100 Bioanalyzer***(or 2200 TapeStation****) is used.

We check total RNA integrity using an Agilent Technologies 2100 Bioanalyzer(or 2200 TapeStation) with an RNA Integrity Number (RIN)*** value greater than or equal to 7.
RNA that has DNA contamination will result in an underestimation of the amount of RNA used. We recommends including a DNase step with the RNA isolation method.
However, contaminant DNA will be removed during mRNA purification It is very important to use high-quality RNA as the starting material.
Use of degraded RNA can result in low yield, over-representation of the 5' ends of the RNA molecules, or failure of the protocol.
***http://www.genomics.agilent.com/CollectionSubpage.aspx?PageType=Product&SubPageType=ProductData&PageID=1648
****http://www.genomics.agilent.com/article.jsp?crumbAction=push&pageId=900109

DNA QC Criteria

Platform	Library Type	Library Kit	Total Amount	DIN	etc
NovaSeq	Amplicon DNA library	Metagenome Amplicon	-	-	Conc > 0.1ng/ul
NovaSeq	Amplicon DNA library	target amplicon DNA	-	-	Conc > 0.1ng/ul
NovaSeq	Modified Library	GBS library(single enzyme)	0.200ug	-	
NovaSeq	Modified Library	GBS library(double enzyme)	0.200ug	-	
NovaSeq	Modified Library	RAD Library(single Library)	-	-	
NovaSeq	Modified Library	RAD Library(double Library)	-	-	
NovaSeq	Modified Library	ETC	-	-	
NovaSeq	Targeted DNA library	SureSelect V6-Post	1.000ug	-	
NovaSeq	Targeted DNA library	SureSelect V6+UTR-post	1.000ug	-	
NovaSeq	Targeted DNA library	SureSelect V5-post	1.000ug	-	
NovaSeq	Targeted DNA library	SureSelect V5+UTR-post	1.000ug	-	
NovaSeq	Targeted DNA library	SureSelect V4-post	1.000ug	-	
NovaSeq	Targeted DNA library	SureSelect V4+UTR-post	1.000ug	-	
NovaSeq	Targeted DNA library	SureSelect Mouse	1.000ug	-	
NovaSeq	Targeted DNA library	SureSelect Human Methyl-seq	1.000ug	-	
NovaSeq	Targeted DNA library	SureSelect Mouse Methyl-seq	1.000ug	-	
NovaSeq	Targeted DNA library	SureSelect Custom	1.000ug	-	
NovaSeq	Targeted DNA library	MBD enriched library	1.000ug	-	
NovaSeq	Targeted DNA library	mt DNA	0.100ug	-	
NovaSeq	Targeted DNA library	SureSelect V7-Post	1.000ug	-	
NovaSeq	Targeted DNA library	Chromium genome Library (Exome capture)	1.000ug	7	
NovaSeq	Whole Genome library	TruSeq Nano DNA (350)	0.100ug	-	
NovaSeq	Whole Genome library	TruSeq Nano DNA (550)	0.200ug	-	
NovaSeq	Whole Genome library	TruSeq Nano DNA (designated insert)	-	-	
NovaSeq	Whole Genome library	TruSeq Nano DNA (LMW)	0.100ug	-	
NovaSeq	Whole Genome library	TruSeq DNA PCR Free (350)	1.000ug	-	
NovaSeq	Whole Genome library	TruSeq DNA PCR Free (550)	2.000ug	-	
NovaSeq	Whole Genome library	Nextera DNA XT	0.001ug	-	
NovaSeq	Whole Genome library	TruSeq DNA Methylation	0.200ug	-	
NovaSeq	Whole Genome library	Accel Methyl-Seq DNA library	0.200ug	-	
NovaSeq	Whole Genome library	TruSeq ChIP-seq library	0.010ug	-	
NovaSeq	Whole Genome library	Chromium Genome library	1.000ug	-	
NovaSeq	Whole Genome library	Nextera mate pair library (gel free)	1.000ug	-	
NovaSeq	Whole Genome library	Nextera mate pair library (gel plus)	4.000ug	-	
NovaSeq	Whole Genome library	Accel-NGS 2S PCR-Free kit (350bp insert)	0.500ug	-	
NovaSeq	Whole Genome library	Accel-NGS 2S PCR-Free kit (550bp insert)	1.000ug	-	

RNA QC Criteria

Platform	Library Type	Library Kit	Type	Total Amount	RIN	rRNA ratio	DV200	etc
NovaSeq	mRNA library	TruSeq mRNA	Total RNA	1.000ug	7	1	-	
NovaSeq	mRNA library	TruSeq mRNA	mRNA	0.100ug	-	-	-	
NovaSeq	mRNA library	TruSeq mRNA	exosomal RNA	0.010ug	-	-	-	
NovaSeq	mRNA library	TruSeq mRNA	cDNA	0.100ug	-	-	-	
NovaSeq	mRNA library	TruSeq mRNA(Insert size)	Total RNA	4.000ug	7	1	-	
NovaSeq	mRNA library	TruSeq stranded mRNA	Total RNA	1.000ug	7	1	-	
NovaSeq	mRNA library	TruSeq stranded mRNA	mRNA	0.100ug	-	-	-	
NovaSeq	mRNA library	TruSeq stranded mRNA	exosomal RNA	0.010ug	-	-	-	
NovaSeq	mRNA library	SMARTer universal low RNA library	Total RNA	0.050ug	7	1	-	
NovaSeq	mRNA library	SMARTer Ultra low input RNA library	Total RNA	0.010ug	7	1	-	
NovaSeq	mRNA library	SMARTer Stranded RNA library	Total RNA	0.050ug	7	1	-	
NovaSeq	mRNA library	TruSeq RNA Access	FFPE RNA	0.100ug	-	-	50%	
NovaSeq	mRNA library	Chromium Single Cell RNA library	Total RNA	-	-	-	-	
NovaSeq	mRNA library	Chromium Single Cell VDJ library	Total RNA	-	-	-	-	
NovaSeq	mRNA library	TruSeq mRNA(Microbe)	Total RNA	3.000ug	7	1	-	
NovaSeq	mRNA library	TruSeq Stranded mRNA(Microbe)	Total RNA	3.000ug	7	1	-	
NovaSeq	mRNA library	SureSelect RNA Direct_Human	FFPE RNA	0.100ug	-	-	50%	
NovaSeq	mRNA library	SureSelect RNA Direct_Human	Total RNA	0.100ug	-	-	50%	
NovaSeq	mRNA library	SureSelect RNA Direct_Mouse	FFPE RNA	0.100ug	-	-	50%	
NovaSeq	mRNA library	SureSelect RNA Direct_Mouse	Total RNA	0.100ug	-	-	50%	
NovaSeq	Small RNA library	TruSeq Small RNA library	small RNA	0.100ug	-	-	-	
NovaSeq	Small RNA library	TruSeq Small RNA library	Total RNA	3.000ug	7	1	-	
NovaSeq	Small RNA library	TruSeq Small RNA library	exosomal RNA	0.010ug	-	-	-	
NovaSeq	Small RNA library	SMARTer Small RNA library	Total RNA	0.050ug	7	1	-	
NovaSeq	Small RNA library	SMARTer Small RNA library	small RNA	0.010ug	-	-	-	
NovaSeq	Small RNA library	SMARTer Small RNA library	exosomal RNA	0.010ug	-	-	-	
NovaSeq	Small RNA library	NEBNext Small RNA library	small RNA	0.100ug	-	-	-	
NovaSeq	Small RNA library	NEBNext Small RNA library	Total RNA	3.000ug	7	1	-	
NovaSeq	Small RNA library	NEBNext Small RNA library	exosomal RNA	0.010ug	-	-	-	
NovaSeq	Total RNA library	TruSeq Total RNA with Ribo-Zero	Total RNA	1.000ug	7	1	-	
NovaSeq	Total RNA library	TruSeq Total RNA with Ribo-Zero(Insert size)	Total RNA	1.000ug	7	1	-	
NovaSeq	Total RNA library	TruSeq Total RNA with Ribo-Zero Bacteria	Total RNA	2.500ug	7	1	-	
NovaSeq	Total RNA library	TruSeq Stranded Total RNA with Ribo-Zero H/M/R	Total RNA	1.000ug	7	1	-	
NovaSeq	Total RNA library	TruSeq Stranded Total RNA with Ribo-Zero H/M/R_Gold	Total RNA	1.000ug	7	1	-	
NovaSeq	Total RNA library	TruSeq Stranded Total RNA with Ribo-Zero H/M/R_Globin	Total RNA	1.000ug	7	1	-	
NovaSeq	Total RNA library	TruSeq Stranded Total RNA with Ribo-Zero Plant	Total RNA	1.000ug	7	1	-	
NovaSeq	Total RNA library	TruSeq Stranded Total RNA with Ribo-Zero Bacteria	Total RNA	2.500ug	7	1	-	
