# Supplementary material for: Genome guided, organ-specific transcriptome assembly of the European flounder (P. flesus) from the Baltic Sea
Source: Sci Data. 2024 Oct 30;11:1184. doi: 10.1038/s41597-024-04004-6 (PMC11525550; doi:10.1038/s41597-024-04004-6)
Supplement: Supplementary file 3 — Table S3 [file 41597_2024_4004_MOESM3_ESM.docx]

| **Sample SRA** | **trimmed** | **STAR** | | **Assembly** | |
| --- | --- | --- | --- | --- | --- |
| SRR11936453 | 44103240 | 37453500 | 84.9% | 34415908 | 91.9% |
| SRR11936454 | 53322304 | 39551288 | 74.2% | 35867845 | 90.7% |
| SRR11936455 | 51288798 | 42266938 | 82.4% | 38339475 | 90.7% |
| SRR11936456 | 46013490 | 37266800 | 81.0% | 36228259 | 97.2% |
| SRR11936457 | 64989588 | 55854588 | 85.9% | 50073662 | 89.7% |
| SRR11936458 | 49362040 | 43881538 | 88.9% | 39269803 | 89.5% |
| SRR11936459 | 40307738 | 35421910 | 87.9% | 31234715 | 88.2% |
| SRR11936460 | 56011808 | 48341592 | 86.3% | 42876585 | 88.7% |
| SRR11936461 | 50296360 | 44920754 | 89.3% | 40652091 | 90.5% |
| SRR11936462 | 50768312 | 44968012 | 88.6% | 40973463 | 91.1% |
|  | a | b | b/a | c | c/b |

Table S3. Number of reads in each sample after trimming, mapping to the reference genome, and mapping to the genome-guided, final assembly. Only reads mapped by STAR to the genome were used to construct the assembly in Trinity, and only these reads were considered for mapping back at the final assembly. Mapping of reads to the assembly was done by bwa, using default parameters, by bwa mem command. Mapping was considered successful if both reads of a pair were mapped.
